# Static Stability and Swim Bladder Volume in the Bluegill Sunfish (*Lepomis macrochirus*)

**DOI:** 10.1093/iob/obad005

**Published:** 2023-02-15

**Authors:** M A Fath, S V Nguyen, J Donahue, S K McMenamin, E D Tytell

**Affiliations:** Department of Biology, Tufts University, 200 Boston Avenue, Suite 4700, Medford, MA 02155, USA; Department of Biology, Boston College, 24 Cummington Mall #606, Boston, MA 02215, USA; Department of Biology, Boston College, 24 Cummington Mall #606, Boston, MA 02215, USA; Department of Biology, Boston College, 24 Cummington Mall #606, Boston, MA 02215, USA; Department of Biology, Tufts University, 200 Boston Avenue, Suite 4700, Medford, MA 02155, USA

## Abstract

Static stability is a property inherent to every organism. More stable bodies benefit from a lower energy cost associated with maintaining a desired orientation, while less stable bodies can be more maneuverable. The static stability of a fish is determined by the relative locations of its center of mass (COM) and center of buoyancy (COB), which may change with changes in swim bladder volume. We hypothesized, however, that fish would benefit from consistent static stability, and predicted that changes in swim bladder volume would not alter the overall pattern of COM and COB locations. We used micro-computed tomography to estimate the locations of the COM and COB in bluegill sunfish (*Lepomis macrochirus*). Using this technique, we were able to find a small but significant difference between the location of the COM and COB for a given orientation. We found that the swim bladder can change shape within the body cavity, changing relative locations of the COM and COB. At one extreme, the COB is located 0.441 ± 0.007 BL from the snout and 0.190 ± 0.010 BL from the ventral surface of the pelvic girdle, and that the COM is 0.0030 ± 0.0020 BL posterior and 0.0006 ± 0.0005 BL ventral to the COB, a pattern that causes a nose-up pitching torque. At the other extreme, the COM is anterior and dorsal to the COB, a pattern that causes the opposite torque. These changes in location seems to be caused by changes in the shape and centroid location of the swim bladder within the body: The centroid of the swim bladder is located significantly more posteriorly in fish oriented head-down. The air in the bladder “rises” while heavier tissues “sink,” driving a change in tissue distribution and changing the location of the COM relative to the COB. Supporting our hypothesis, we found no correlation between swim bladder volume and the distance between the COM and COB. We conclude that bluegill are statically unstable, requiring them to expend energy constantly to maintain their normal orientation, but that the pitch angle of the body could alter the relative locations of COM and COB, changing their static stability.

## Introduction

**Fig. 1:**
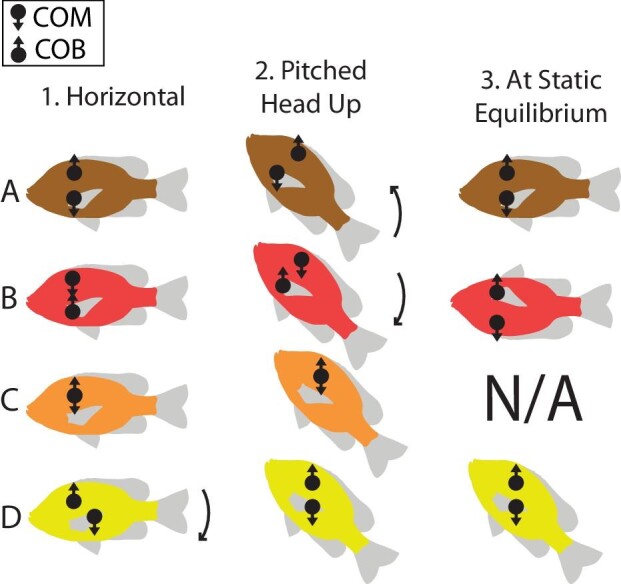
Four possible configurations of the COM and COB that lead to different static equilibria. Panels A–D show fish with different relative locations of the COM and COB. The Columns 1–3 show each fish in 3 orientations: horizontal (column 1), pitched 45° head up (2), and at static equilibrium (3). The gravitational and buoyant force vectors are indicated on the COM and COB, respectively. If the COM and COB are not vertically aligned, this generates torques, indicated with a curved arrow. Although all fish have a state of static equilibrium, only fish A is statically stable in a horizontal, dorsal-side-up orientation.

Fish are remarkably stable, with most fish maintaining a level orientation with their dorsal side up, despite strong and unpredictable forces from the environment. But stability in fish is complex, and it depends on whether a fish is swimming, maneuvering, or sitting still in the water column (Weihs 1993; Webb 2005; Webb and Weihs 2015). One aspect of stability—static stability—underlies all the others because it arises from the intrinsic torques that develop through the interaction of gravity and buoyancy via the heterogeneous distribution of mass and volume throughout the fish body. Depending on the distribution of denser and lighter tissues within a fish's body, gravity and buoyancy might combine to produce torques that would tend to right the fish following a perturbing force (Fig. 1A). A fish like this would be statically stable, and would not need to expend energy to maintain its normal orientation, but would be less maneuverable (Webb 1997). Conversely, but likely more common for fish (Harris 1936; Aleyev 1977; Webb and Weihs 1994; Fish and Holzman 2019), with a different distribution of tissue density, gravity and buoyancy could generate torques that would turn the fish away from an upright or preferred orientation, making it less stable (Fig. 1B, C). Such a fish would constantly have to exert energy to maintain orientation, but would be more maneuverable (Webb 1997). In this way, the distribution of tissues with different densities throughout an organism can predispose its body toward being more stable or more maneuverable before considering other morphological, physiological, or behavioral adaptations to enhance those properties.

In physical terms, the static stability of any submerged object, including fish, is ultimately determined by two forces: gravity and buoyancy. The relative magnitude of these forces determines whether an object floats or sinks, but the balance of these forces on the object determines its stable orientation (Roth 2013). To predict that orientation, the gravitational and buoyant forces can be treated as acting at discrete points in the object. The buoyant force acts upward on the center of buoyancy (COB), a point located at the geometric center (also called the centroid) of the displaced fluid volume (Smits 2000). The gravitational force pulls downward at a location called the center of mass (COM), which can be estimated by dividing the object up into many small elements and taking the average location of all the elements, weighted by their mass (Walker 2015). The location of the COM is thus determined by the distribution of mass throughout a fish and the COB is determined by the distribution of volume, and they can have different locations if these distributions are not uniform. If the COB is located directly above the COM, the fish would be in a stable equilibrium (Fig. 1A); that is, small perturbations causing a misalignment of the two points will result in torques which act to move the COB back above the COM. If the COB is located directly below the COM, the fish is in an unstable equilibrium; there is no torque in this orientation, but any small perturbation would tend to produce a torque that would move the fish away from that equilibrium (Fig. 1B). If the COB and COM are both in the same location, the fish is neutrally stable, and any orientation is an equilibrium (Fig. 1C).

Beyond simple physics, fishes also have a desired orientation that may or may not align with the orientation of static equilibrium. There are not a large number of measurements of the locations of COM and COB for fish, but the evidence suggests that the normal preferred orientation is not statically stable. Fishes often roll or pitch from the preferred dorsal-side-up orientation to a side or “belly up” orientation when anesthetized or dead, indicating that the dorsal-side-up orientation is unstable (Harris 1936; Aleyev 1977; Webb 2005). Aleyev (1977) found horizontal displacements between the COM and COB in many fish, indicating a pitching torque. More recently, Webb and Weihs (1994) found no significant difference between the mean longitudinal locations of the COM and COB in rock bass, perch, bluegill, and eel, although this may have been due to the difficulty in accurately resolving these locations.

If fish are statically unstable, it means that they must constantly spend energy maintaining their desired orientation. Webb (2002) suggests that stabilization costs play a large role in energy expenditures. The benefit of instability, though, is that fish that are statically unstable may be able to turn or maneuver more rapidly, because the passive action of gravity and buoyancy could aid in maneuvering (Weihs 1993; Webb 2005; Webb and Weihs 2015).

Further complicating the relationship between the location of the COM and COB in fish is the presence of the swim bladder. The swim bladder is a gas-filled organ, which is much less dense than the rest of the fish (Alexander 1966). Fish can add or remove gas to the bladder in response to changes in depth and pressure (Steen 1970). Changes in swim bladder volume could affect the distribution of volume and mass throughout the fish. If the changes in volume and mass result in movement of the COM or COB, then a change in swim bladder volume could also alter static stability, potentially changing a statically stable fish into an unstable one, or vice versa.

Previously, Webb and Weihs (1994) studied the location of the COM and COB in bluegill and other fish. They concluded that there is no significant difference between the two points, that the inclusion or exclusion of the swim bladder does not change stability, and that the redistribution of swim bladder volume is unlikely to significantly affect the pitching equilibrium of these fish. In this study, we aimed to validate a new method to determine the locations of the COM and COB in bluegill sunfish (*Lepomis macrochirus*), and to examine whether the COM and COB locations change when the swim bladder changes volume. We used micro-computed tomography (µCT) to estimate the location of the COM and COB in fish with untreated swim bladders and in fish with air removed from the swim bladder. Following Webb and Weihs (1994), we hypothesized that it would be beneficial for fish if their static stability does not change when the swim bladder changes volume, because this would simplify the neural control of orientation. For static stability to stay the same, changes in swim bladder volume would have to be uniformly distributed relative to the COM, which would allow the swim bladder to change volume without affecting the relative location of the COB relative to COM.

## Methods

### Animal preparation and scanning

We collected 15 bluegill sunfish (*L. macrochirus*) with a seine from the littoral zone of White Pond in Concord, Massachusetts, in the summer of 2019. The bluegill sunfish was chosen because it is one of a few fish whose COM and COB locations have been previously reported (Webb and Weihs 1994), allowing for the comparison of the µCT measurements with traditional ones. Prior to scanning, the fish were held in 10 gallon tanks for at least 2 months and fed five days a week. The water in the tank system was filtered with mechanical and carbon filters. Fish were cared for, handled, and euthanized according to IACUC protocol M2021-99. Fish were euthanized using an overdose of buffered MS-222 (Acros Organics) solution (0.04%) in 1 L water. Before being scanned, animals were massed and the body length and standard length were measured. Here, we will refer to the standard length as 1 BL. The caudal fin rays often bent while the fish was being scanned. Measuring from the snout to the beginning of the caudal fin rays provided a more reliable measurement of length when comparing between the fish and its reconstructed model.

To generate a range of swim bladder volumes, we used a syringe to add or subtract air from the swim bladder. We estimated swim bladder volume using the relationship from McNabb and Mecham (1971) that the volume of the swim bladder is 8.87 mL per 100 g body weight. Based on this, we removed approximately 25% of air in the swim bladder using a hypodermic needle from seven fish. We also attempted to insert air into the swim bladder. However, the injected air would most often escape the over pressurized bladder through the insertion site and float freely through the body cavity. Only one fish retained the air in the swim bladder and not in the body cavity. Six control fish were left untreated. Once treated, the fish were preserved in ethanol to prevent any bloating that might occur between the time of euthanizing and final scanning (between 1 and 5 days). Fish were placed in increasing concentrations of EtOH (12.5, 25, and 50%) at equal intervals over the course of 2 days.

**Fig. 2:**
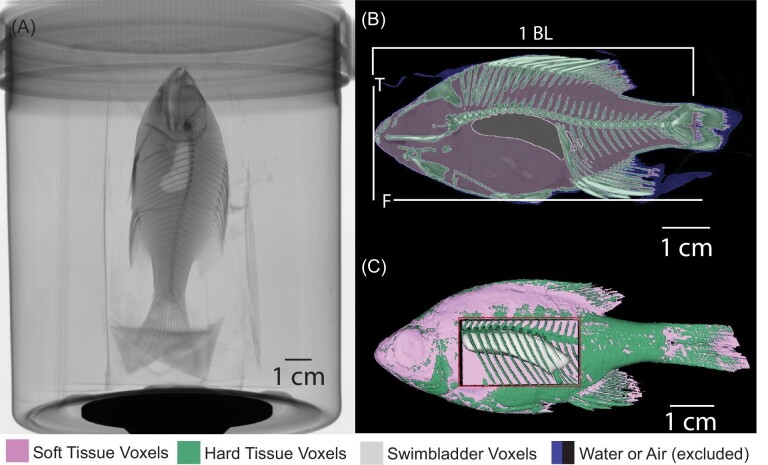
(A) Fish were µCT scanned to calculate the locations of the center of mass and center of buoyancy. The panel shows a 2D planar X-ray image of a fish, taken just before scanning. (B) A sample of a slice through a reconstruction. T indicates the transverse slice we used as the front of the fish: the mandibular symphysis. F indicates the frontal slice through the ventral minimum point on the fish: the ventral-most point of the articulation between the pelvic girdle and pelvic fin. We used the standard length for body length, measured as the distance from the front of the fish to the base of the caudal fin rays. Voxels are colored by tissue category. Water (blue) and air (black) were removed for the analysis. (C) A 3D reconstruction. A red window indicates the swim bladder.

The fish were µCT scanned (Fig. 2) to generate 3D reconstructions from which COM and COB locations were calculated. Fish were placed in a plastic Ziplock bag with about 3 mL of 50% ethanol solution. The bag was then placed into a small plastic container and radiotransparent foam was added to prevent the fish from moving during the scan (Fig. 2A). µCT scans are most accurate when the thickness of the specimen that the X-ray must penetrate does not change as the specimen rotates in the scanner. To minimize changes in thickness due to rotation, 11 fish were placed in the scanner vertically with their heads pointing down. An additional four fish were scanned head down. Fish were scanned no later than one week following fixation using a SkyScan 1275 µCT scanner (Bruker, Kontich, Belgium) at a resolution of 0.0483 mm with an X-ray source voltage of 45 kV and current of 222 mA. Projection images were generated over 360° with a 0.1° rotation step and 6 averaging frames. During reconstruction of the scans, thresholding, ring artifact reduction, dynamic range, and beam hardening correction values were consistent across all scans using NRecon (Bruker, Kontich, Belgium). We measured the horizontal (X) location of the COM and COB as the distance from the tip of the mandible and the vertical (Z) location as the distance from the location where the pelvic fins meet the body (Fig. 2B).

### Density calibration

We used the intensity at each voxel as a measure of density. Voxel intensity was determined using NRecon (Bruker, Kontich, Belgium), converting the attenuation coefficient from the scan into 8-bit integers. Here, we present intensity measured as values between 0 (all X-rays pass through) and 1 (all X-rays absorbed). Voxel intensity reflects the X-ray absorption, at a given voxel. The amount of X-ray absorption is determined by density, but it is also influenced by chemical composition at that point (Du Plessis et al. 2013). For homogeneous objects, digital determination of COM location is more accurate and shows less variability between estimates than the plumb line technique (Macaulay et al. 2017). While this is true for an object of uniform composition, voxel intensity does not necessarily scale linearly across materials with different chemical compositions.

Following recent studies (Durston et al. 2022), we estimated a scaling relationship for hard tissues (bones and scales) and a separate scaling factor for soft tissues (muscle, viscera, fat, etc.). Tissues were identified using morphological location and voxel intensity. To create a scaling relationship for hard tissue, we included a 5 mm phantom (QRM-MicroCT-HA D4.5, serial number MHA-192, Möhrendorf, Germany). The phantom has cylindrical inserts containing four known densities of calcium hydroxyapatite (1.16, 1.25, 1.64, and 1.90 g/cm³). We isolated each concentration of hydroxyapatite in the phantoms and recorded the average voxel intensity and compared it to the density of the sample. To create a scaling relationship for soft tissues, using 3D Slicer (Version 4.11, Fedorov et al. 2012), we compared the voxel intensity and density of two soft tissues: anterior dorsal epaxial muscle and the lens of the eye. These two tissues were chosen because they have different densities, different gray values, and they can be easily isolated in a scan. For each scan, we recorded the average voxel intensity of a sample of the dorsal epaxial muscle and the one of the eye lenses. Then, using five fish which were not scanned, we measured the density of dorsal epaxial muscle and lens tissue. We isolated two samples of dorsal epaxial muscle as well as the lens from each eye of the five fish. We weighed each tissue sample to get the mass and we measured the amount of water the sample displaced to get the volume. We divided the sample's mass by its volume to calculate the density.

### COM and COB location calculation

We calculated the COM and COB locations for 13 of the 15 bluegill reconstructions. This excludes two fish with free floating air in the body cavity (both scanned head down) from the effort to increase the volume of the swim bladder. We used the reconstructed virtual fish models to calculate the location of the COM and COB. If there was any bending of the fish in the scan, the fish was digitally straightened using unwind.exe (Williams et al. 2020). This process also guaranteed that the fish were oriented along the same X, Y, and Z coordinate system. We then used 3D Slicer (Version 4.11, Fedorov et al. 2012) to isolate only the voxels that comprised the fish, removing any voxels including support material or background.

Custom Matlab software (R2020b; Mathworks, Inc, Natick, MA) was used to calculate the locations of the COM and COB from the isolated fish reconstruction. The COB was calculated by determining the geometric center of the isolated fish volume


\begin{eqnarray*} {\rm{\ }}{{{\bf x}}}_{COB} = \frac{{\mathop \sum \nolimits_{i,j,k} {{{\bf x}}}_{ijk}{F}_{ijk}}}{{\mathop \sum \nolimits_{i,j,k} {F}_{ijk}}}\ , \end{eqnarray*}

where **x***_ijk_* = [*x_i_ y_j_ z_k_*] is the 3D location of the voxel with indices $i,\ j,\ $and *k*, and ${F}_{ijk}$ is 1 for voxels inside the fish's volume and 0 otherwise.

The COM was calculated as the average location in the body, weighted by density:


\begin{eqnarray*} {\rm{\ }}{{{\bf x}}}_{COM} = \frac{{\mathop \sum \nolimits_{i,j,k} {{{\bf x}}}_{ijk}{\rho }_{ijk}}}{{\mathop \sum \nolimits_{i,j,k} {\rho }_{ijk}}}, \end{eqnarray*}

where ${\rho }_{ijk}$ is the density of the voxel at indices $i,\ j,\ $and *k*. Different scaling relationships were used to determine the density of each voxel for hard and soft tissue (see “Density calibration”).

**Fig. 3:**
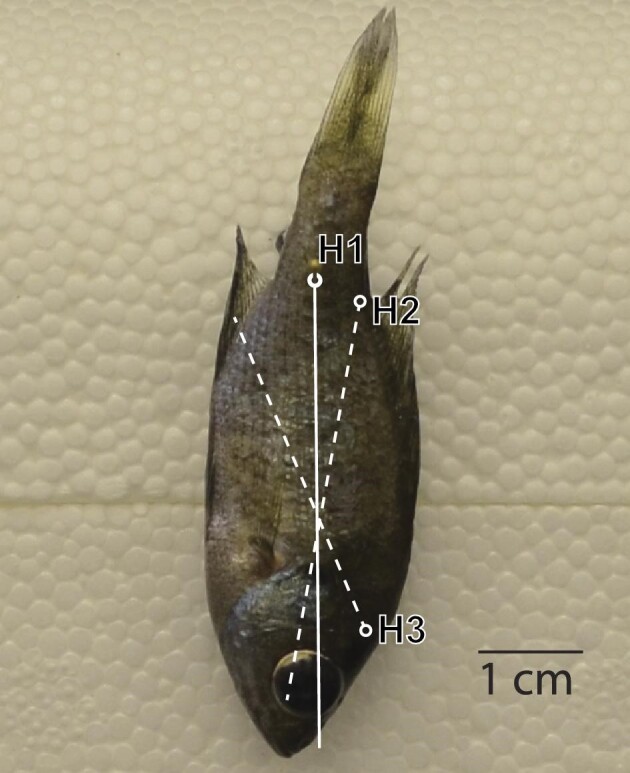
The measurement of the center of mass via the plumb line technique. Shown here is an image of a fish hanging from suspension point H1. A line perpendicular to the gravitational vector that goes through H1 is the solid white line. H2, H3, and their associated dotted lines represent the two other suspension points and their associated vertical lines and have been overlaid on the H1 image. The COM is located at the intersection of these lines.

We measured the location of the COM of a subsample µCT scanned fish (*n* = 4) using the plumb line technique (also called the suspension method; Fig. 3). All fish selected for the plumb line measurements were µCT scanned in the same orientation. The plumb line technique locates an object's COM by that object and a weighted string (plumb line) from the same point of rotation. The object will rotate until its COM is below the point of rotation and the plumb line is used to identify a line on the object. The object is then suspended from a new point of rotation and the plumb line is used to identify a second line. The intersection of those lines indicates the object's COM. We pierced each fish with a dissecting pin and hung the sample so that it could rotate freely and then photographed the fish in a lateral view. A weighted plumb line was suspended and included in the image to indicate the gravity vector. This process was repeated two more times from two different points of rotation, a total of three times for each fish. Instead of using a string, we used Photoshop (2021, Adobe, Venture, CA) to draw a digital plumb line through the suspension point parallel to the gravitational vector. We repeated drawing process for each imaged suspension point. The three images were digitally rotated so that the fish was aligned (Fig. 3). The three lines drawn from the hanging point on each image intersected at three points, each representing one estimation of the COM from a pair of hangings. The average location of these points was taken as the center of mass.

### Swim bladder centroid determination

We used 3D Slicer to segment the swim bladder voxels. Using the same equations presented above to determine the geometric centroid (COB) of the fish, we calculated the location of the centroid of the swim bladder. We were then able to compare the location of the swim bladder centroid in fish scanned in the head-up and to the swim bladder centroid location in fish scanned in the head-down orientation. This analysis included two fish, which were not used elsewhere in the analysis: these individuals contained air bubbles in the mouth or gut, which would affect COM calculations and were therefore excluded from other analyses. However, these bubbles would not affect the shape of the swim bladder, so they were included in the swim bladder centroid analysis.

### Statistical analysis

Most measured values are reported as mean ± standard deviation. Tissue density and voxel intensity are presented as mean ± standard error. We estimated regressions for the density and mass relationship as well as the swim bladder and COM/COB location relationships with a least-squares regression. We used a one-sample *t*-test to measure if the difference between the measured mass of the fish and the estimated mass of the fish was different than zero. We used pairwise *t*-tests to determine if there were significant differences between (1) the locations of the COB and the COM as calculated from the µCT scan reconstruction, (2) the locations of the COB and the COM as determined by the plumb line technique, and (3) the location of the COM as determined from µCT scan reconstruction and the plumb line technique.

## Results

### Density estimation

**Fig. 4:**
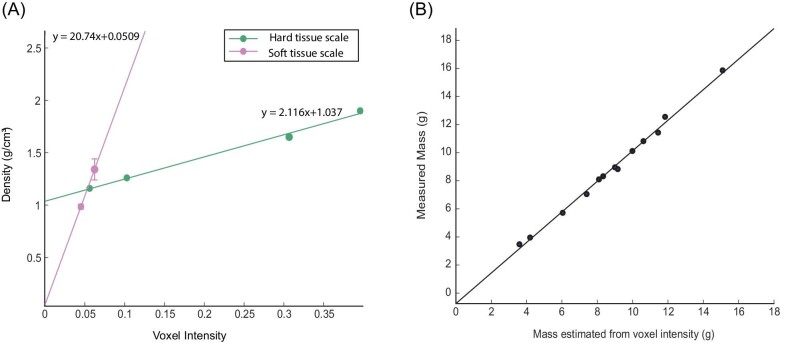
Density scaling and mass calculation. (A) The scaling relationships for voxel intensity and density for hard tissue and soft tissue. The soft tissue regression was established with muscle and lens tissue from the fish. The hard tissue regression was established using inserts from a hydroxyapatite phantom included in the scan. Error bars indicate standard error. If they are not visible on the plot, they are presented in Table 1. (B) The measured mass of a fish and the mass of the same fish as estimated from the µCT scan reconstruction.

We measured the density of muscle and lens, as well as the voxel intensity for muscle, lens, and four concentrations of hydroxyapatite (Table 1). These values yielded the scaling relationships presented in Fig. 4A. To determine if these relationships were accurate, we used them to estimate density of each voxel for a given fish. We then added the mass of each voxel to estimate the total mass of the fish. We then compared the estimated mass to the measured mass (Fig. 4B). Using scaling relationships for hard and soft tissues, we found that voxel intensity could be used to accurately estimate the mass of the fish (*r*^2^ = 0.9955). The difference in the estimated and true mass was not significantly different from zero (*t* = –0.33, *P* = 0.78), indicating a 1:1 relationship between the actual mass and estimated mass.

**Table 1:** Density and voxel intensity information for samples used to establish scaling relationships

	Tissue	Density (g/cm³)	Volume measured (cm³)	Voxel intensity	Sample volume (cm³)
Soft tissue	Muscle	0.98 ± 0.02	0.19 ± 0.02	0.045 ± $9 \times 10^{-8}$	2.4 ± 1.6
	Lens	1.3 ± 0.1	0.004 ± 0.001	0.062 ± $1 \times 10^{-5}$	0.004 ± 0.001
Hard tissue	HA Insert 1	1.16	NA	0.056 ± 0.003	0.009 ± 0.002
	HA Insert 2	1.26	NA	0.10 ± 0.01	0.010 ± 0.002
	HA Insert 3	1.65	NA	0.31 ± 0.02	0.013 ± 0.004
	HA Insert 4	1.9	NA	0.40 ± 0.06	0.016 ± 0.007

Note. Density and voxel intensity values reported as mean ± standard error.

We measured the location of the COM using both the digital and hanging techniques in four individuals. For repeated measurements taken from the same individual, the measured COM location varied by 2 ± 2% BL. The COM locations calculated from the μCT scan reconstructions were 3 ± 3% BL from the COM locations determined by hanging on the same fish, within the range of precision of the plumb line technique.

### The swim bladder and the location of the COM and COB

The bluegill individuals (*n* = 15) we measured ranged from 3.48 to 15.86 g with a mean mass of 9.3 ± 3.5 g. The standard lengths of the fish measured from 6.50 to 10.4 cm with a mean standard length of 8.38 ± 0.01 cm. The volume of the fish ranged from 3.83 to 15.58 cm³ with a mean volume of 9.2 ± 3.2 cm³.

**Fig. 5:**
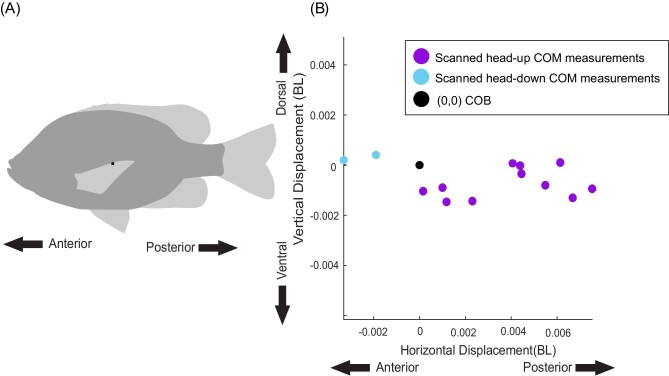
The calculated locations of the COM relative to the COB for all fish measured. (A) A bluegill with the average location of the COB marked with a black square. (B) All COM measurements from 13 bluegill plotted relative to the COB from the same fish. COB is thus at (0,0) for each individual. The plot is to scale with the black square in panel A.

We were able to measure the location of the COM and COB in 13 of the 15 fish. Two were excluded because of air in the body cavity. In most fish, the COM was located posterior and ventral to the COB (Fig. 5). On average, the COM was located 0.442 ± 0.008 BL from the snout and 0.193 ± 0.010 BL from the ventral surface, and the COB is located 0.441 ± 0.007 BL from the snout and 0.190 ± 0.010 BL from the ventral surface (Fig. 5A). Comparing the location of the COM relative to the COB in each individual, we found that the COM is significantly posterior (*P* = 0.008) and ventral (*P* = 0.009) to the COB (Fig. 5B). For fish scanned head-up in the scanner, the COM is 0.0030 ± 0.0020 BL posterior and 0.0006 ± 0.0005 BL ventral to the COB on average. This arrangement would create a head-up pitching torque, which was 3 ± 3 μN·m.

We also measured the location of COM using the plumb line technique (Alexander 1983) in 4 out of the 13 fish scanned. Each fish was hung from 3 points, resulting in 3 estimates of the location of the COM. The average distance between the three points was 0.023 ± 0.029 BL. The location of the COM was found to be 0.439 ± 0.001 BLs from the snout and 0.1957 ± 0.0006 from the ventral minimum. We found no significant difference between the COM as determined by the plumb line technique and by the µCT method in the vertical (*P* = 0.28) or horizontal (*P* = 0.78) directions. Based on plumb line measurements (unlike our µCT measurements), we were not able to detect a difference between the locations of the COM and COB in the vertical (*P* = 0.27) or horizontal (*P* = 0.78) direction.

**Fig. 6:**
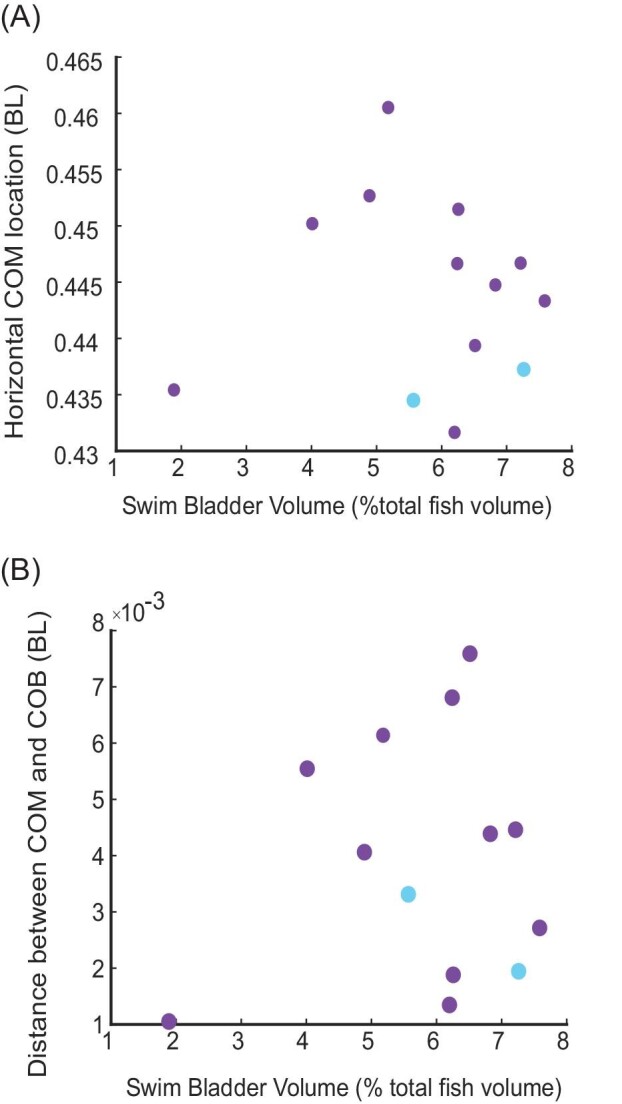
Differences in swim bladder volume do not affect the location of COM or the distance between it and COB. (A) Horizontal location of the COM compared to the volume of the swim bladder. (B) Total distance between COM and COB relative to swim bladder volume.

We found no correlation between the volume of the swim bladder and the location of the COM for all fish (*P* = 0.76, *R*^2^ = 0.008, Fig. 6A). Fig. 6A shows the relative locations of the COM and COB in fish with swim bladder volumes ranging from 1.9 to 7.6% total fish body volume. In untreated fish, the mean swim bladder size is 6.2 ± 1.2% of the total body volume (range from 4.0 to 7.6% body volume). We were able to remove air from seven individuals. The mean swim bladder volume for fish with air removed was 4 ± 1% total fish body volume. The swim bladder was 7.2% total fish body volume in the individual with air added to the bladder.

We also found no significant relationship between swim bladder volume and the total distance between the COM and COB (Fig. 6B) (*P* = 0.67, *R*^2^ = 0.0162). Further, we did not find any correlation between the swim bladder volume and the horizontal (*P* = 0.94, *R*^2^ = 0.0058) or vertical (*P* = 0.41, *R*^2^ = 0.06) components of the COM/COB displacement.

**Fig. 7:**
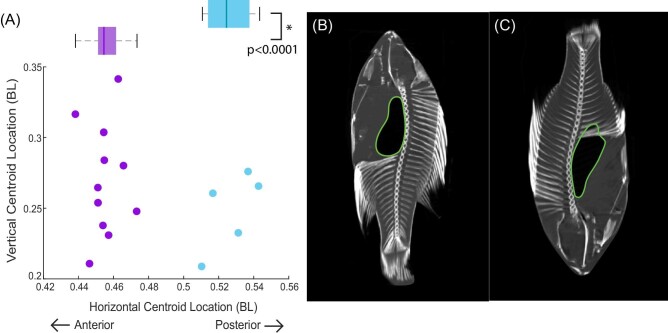
The location of the centroid of the swim bladder in bluegill sunfish depends on the fish's orientation in the scanner. (A) The centroid location in fish scanned head up (purple) and head down (pink). (B) A fish placed in the scanner head up. (C) A fish placed in the scanner head down. The swim bladder is outlined in green.

Although the swim bladder volume does not affect the location of COM or COB, the swim bladder location does have an effect on COM. In fish scanned head up, the centroid of the swim bladder is positioned significantly (*P* < 0.001) more posteriorly than in fish scanned head-down (Fig. 7). On average, the centroid of the swim bladder in fish scanned head-down (*n* = 4) is located 0.53 ± 0.01 BL from the snout. The centroid of the swim bladder in fish scanned head-up (*n* = 11) is located 0.45 ± 0.01 BL from the snout. In all fish, the swim bladder is just ventral to the vertebrae. In fish scanned head up, the bladder extends from posterior to the neurocranium and terminates before the first haemal/interhaemal arches. In fish scanned head-down, there is a larger distance between the posterior edge of the cranium and the bladder and the bladder straddles the first haemal/interhaemal arches.

## Discussion

We estimated the location of the COM and COB for bluegill sunfish with µCT data, and compared our measurements to previous ones (Webb and Weihs 1994). The µCT-based method is at least as accurate as previous techniques for estimating the COM, and because of the accuracy of the µCT scan, it is more accurate at determining the location of the COB. Similar to Webb and Weihs (1994), we found that the COM is very close to the COB; however we were able to detect a significant difference between the two points indicating that the fish is not neutrally stable, but statically unstable (i.e., like Fig. 1D, not Fig. 1C). We also found that location of the COM could be posterior or anterior to the COB depending on the orientation of the fish. Supporting our hypothesis, and in agreement with Webb and Weihs (1994), we also found that swim bladder volume did not affect the distance between COM and COB, suggesting that the static stability of fish does not change as they ascend or descend in the water column.

### Calibrated digital COM measurements are at least as accurate as the plumb-line technique

The extremely small distances between the COM and COB have made it difficult to determine the relative location of the two points. Earlier studies in fishes have measured the location of COM and COB using different techniques such as serial sectioning, balancing, digital modeling, and the plumb-line (suspension) technique (Aleyev 1977; Webb and Weihs 1994; and Fish and Holzman 2019). These measuring techniques can introduce error. Serial sectioning results in loss of water and tissue as the fish is sliced and limits COM location determination to one axis. Traditional plumb line techniques require the creation of a physical model to locate the COB. Differences in shape or 3D orientation of the model or fish while measuring can lead to differing location measurements. These limitations are not trivial. Where it is reported, repeated measurements of the location of the COM and COB locations in fish varied by ±2% body length, which is greater than the distance between the two points (Webb and Weihs 1994). Similarly, we found that the average distance between points measured by the plumb line technique was 2.3 ± 2.9% body length.

Here we used µCT to estimate the locations of the COM and COB. Studies reporting on homogeneous objects have shown that using a µCT scan to digitally locate the COM of the object can be more accurate than using the plumb line method (Macaulay et al. 2017). CT or MRI scans have been used to find the COM in humans (Pearsall et al. 1996), dogs (Amit et al. 2009), and birds (Durston et al. 2022). However, µCT scans also have limitations in measuring the location of the COM, because animals are not made of homogeneous materials. Using voxel intensity derived from the attenuation coefficients from µCT scans to calculate the COM is one source of error. Voxel intensity is a measure of X-ray absorption, which is largely determined by density, but can also vary be effected by other factors such as X-ray scattering and chemical composition. Our study, like others (Durston et al. 2022), accounted for this by calibrating scans with samples of specific tissues of known densities.

We used separate scaling factors for hard and soft tissues to calibrate our scans and account for non-density-related changes in X-ray absorption. It can be noted that when we tried other voxel intensity/tissue density relationships (using raw, un-calibrated voxel intensity, or using a mean density value for soft tissue and one mean value for hard tissue), measurements of the COM and COB locations were of the same magnitude and direction as the calibrated measurements. However, here we use the calibrated measurements as that have been shown to be the most accurate (Durston et al. 2022). Using this calibration, we were able to estimate the body mass within 2.4 ±3.8% from the reconstructed scans. We also compared the location of the COM determined from calibrated µCT scan to the plumb line method. We found that on average, our digitally determined location was 3% BL from the average measured plumb line COM location. This is comparable to the variability from repeated measures of ± 2% body length reported by Webb and Weihs (1994). We conclude that using calibrated µCT scans to calculate the location of the COM for a fish is at least as accurate at determining the COM location as traditional techniques.

Using µCT scans has an additional advantage: They provide a precise reconstruction of fish volume and body shape, so they can be used to estimate COB location with extreme accuracy. Use of the plumb line technique requires the creation of a separate animal model made from a homogeneous material; Aleyev (1977) used wood, Webb and Weihs (1994) used plaster, and Fish and Holzman (2019) used a digital model to determine the COB. Then researchers would compare measurements between the model and the actual animal. The CT-based method uses the same digital model to calculate both COM and COB, thus removing error introduced by comparing the fish to a separate model.

We found no statistical difference between the location of the COM as determined by the plumb line technique and the CT scan method, indicating that using either technique may be sufficient for locating the COM. The plumb line technique requires less time and resources. However, it is less accurate than the CT method, and it is particularly complex to locate the COB relative to COM using plumb line techniques. For example, Webb and Weihs (1994) found no statistical difference between the COM and COB in the bluegill sunfish and concluded that the fish is neutrally stable in pitch and roll. However, using µCT we were able to detect a significant difference between the location of the COM and COB for a given orientation as the COM was located more consistently in the same place relative to the COB. Like the CT method, the plumb line technique requires hanging the fish in different orientations, which may allow internal tissues to shift, potentially altering the measured location of the COM.

### Static stability does not change with depth or swim bladder volume

The swim bladder of many fish changes volume in close accordance with Boyle's law (Jones 1951), thus changing the distribution of tissues within the fish as the fish changes depth and hydrostatic pressure changes. Bluegill can be found in shallow or deeper waters, as the fish tends to move offshore after sunset and nearshore after sunrise (Baumann & Kitchell 1974). We measured swim bladders with a range of volumes of 5.7% total fish volume, roughly equivalent to a bluegill swimming from a depth of 1 m down to about 3.8 m in fresh water. Other fish undergo more extreme vertical migrations; it is unclear how changes in swim bladder volume may affect the stability of these fish.

We did not find any correlation between swim bladder volume and the distance between the COM and COB in bluegill, indicating that there is no change in the stable equilibrium orientation as the fish changes depth. This supports our hypothesis that changes in swim bladder volume would be distributed evenly relative to the center of mass, which would maintain the same stable equilibrium. Moreover, to maintain a constant buoyant force as they change depth, fish will try to maintain a constant swim bladder volume, regardless of depth (Moreau 1876), which will also tend to maintain static stability.

While this is true in bluegill, the relationship between swim bladder volume and stability needs to be explored in other fish. Swim bladder morphology and function vary dramatically across fish species (Pelster 2011). Bluegill are physoclistic: they do not have a connection between the gut and swim bladder. Other (physostomic) fish have a connection between the esophagus to either a gas bladder or lungs, which can allow the fish to gulp air to fill the organ (Graham 1997). Physostomic fish may thus experience larger or faster changes in swim bladder volume. The morphology of the body cavity, which also varies in different fish species, constrains the shape changes of swim bladder in different fish (Webb and Weihs 1994). Additionally, the morphology of the swim bladder in other fish can be different from bluegill, such as the double chambered swim bladder various cyprinids (Robertson et al. 2008; Pees et al. 2010). Finally, while there is no evidence that bluegill can control the shape of the swim bladder as it changes volume, some fish may be able to. In zebrafish, the anterior chamber of the swim bladder can be deflated under the control of the sympathetic nervous system (Dumbarton et al. 2010). Thus, future researchers should examine the locations of COM and COB in fish with different swim bladder types and body shapes to determine if stability tends to stay the same in fish as the swim bladder changes volume across fish with varied morphologies.

### COM and COB locations may change with body orientation

We did not see a change in equilibrium orientation with swim bladder volume, however, we did find that the COM and COB change location relative to one another for fish at extreme pitch angles. A bluegill is unlikely to spend much time at these angles, but they represent the range of possibilities. For all the fish scanned in a head-up orientation, the COM was posterior to the COB, generating a head-up pitching torque. In 9 out of 11 head-up scans, the COM was ventral to the COB resulting in no rolling torque. Conversely, fish (*n* = 2) placed in the scanner head-down had a COM located anterior and dorsal to the COB, a configuration which would result in a head-down pitching torque and a belly up pitching torque. The opposing COM/COB configurations in fish scanned head up (COM anterior and ventral to COB) and head down (COM anterior and dorsal to the COB) result in different stable equilibrium orientations. In both cases, the COM is below the COB, making the fish relatively stable in each orientation. The change in relative locations of the COM and COB results from the redistribution of tissues. Air rises upward within the swim bladder to occupy the highest point available, while the heavier soft tissues “sink” downward to occupy lower points in the gut cavity. The centroid of the swim bladder in fish scanned head up is located 0.08 BL more anteriorly compared to the swim bladder centroid of fish scanned head-down. The location of the COM is known to be dynamic, and can move as the body and limbs extend (Alexander 1983). Our observations suggest that the location of COM may also move as tissues redistribute at different pitch angles.

While a pitch-dependent dynamic COM/COB relationship was seen in the dead fish we scanned, the degree to which the bladder is mobile in living fish remains unclear. A number of variables including handling, postmortem physiological changes, tissue property changes associated with chemical fixation, and/or time spent in storage at extreme angles may have facilitated the movement of tissues. If the swim bladder is equally mobile in live fish, then there may be multiple angles at which the COM and COB are vertically aligned, thus producing no destabilizing pitching or rolling torques. Visualizations of the swim bladder in live fish and µCT scans of fish at a horizontal orientation would be necessary to determine the *in vivo* swim bladder mobility and resulting changes to stability.

### Static (in)stability and implications maneuverability and for dynamic stability

This study, along with others examining the COM and COB in bony fish (Aleyev 1977; Webb & Weihs 1994; Fish & Holzman 2019), sharks (Harris 1936, 1938), and cetaceans (Fish 2002) indicate that aquatic animal bodies tend to have COM and COB locations that make the organism unstable or neutrally stable. Since these relative locations generate torques that increase instability, these organisms can change their orientation more easily. Webb (2004) defines “maneuver” as a change in state (direction, position, etc.) for a specific purpose. Maneuverability, often measured in turning ability, helps organisms catch prey or avoid predators (Howland 1974; Domenici 2001; Fish et al. 2003) as well as move successfully through environments (Schrank et al. 1999; Davis et al. 2001; Howe & Astley 2020). The wide array of species that are statically unstable suggests that there may be a selective benefit to instability. Stability and maneuverability are tradeoffs (Weihs 1993; Webb 2005; Webb & Weihs 2015), so one possibility is that the benefit of higher maneuverability outweighs the metabolic cost of instability for many fish species.

Statically unstable fish must use fin and body motions to achieve dynamic stability: a steady state achieved through a set of characteristic state variables (Full 2002). Swimming animals can make powered fin movement to stabilize but they can also take advantage of trimming forces over fins with locations and morphologies (Fish 2002; Harris 1936, 1938). In this way, they take advantage of environmental forces and minimize the need for powered stabilizing motions. However, hovering or slow swimming also presents challenge for stability (Webb 1993, 1997, 2002; Videler 1993), as powered fin motions are the only option for generating stabilizing torques and forces.

Based on our data, maintaining dynamic stability while hovering at a horizontal orientation would require that bluegill generate a constant average torque of 4.46 ± 4 µN·m to counter a head up pitching torque. This torque can be easily counteracted by fin movements. To illustrate this, we estimated the largest possible torque. The largest distance between the COM and COB was 0.74 mm, and the largest buoyant force was 0.081 N. Assuming the moment arm is perfectly perpendicular to the pull of gravity, the largest possible destabilizing torque is 113 µN·m, which is more than 20 times greater than the average. Drucker and Lauder (1999) estimated that bluegill can produce up to 24 mN of horizontal force with the pectoral fins on the upstroke. If we assume that the center of pressure of the fin, where the force is centered, is located approximately 1.2 cm from the COM, then the fin can generate a torque of 288 µN·m, much more than the torque resulting from the buoyant force. The median fins are also effective stabilizers, contributing to both steady swimming and maneuvering (Drucker and Lauder 2001; Standen 2005). Drucker and Lauder (2001) found that the dorsal fin of these fish can produce up to 11.2 mN of laterally directed force and 13.2 mN of posterior directed force. While these forces are smaller than those generated by the pectoral fins, they are still sufficient to generate enough counter pitching torque to maintain a horizontal angle. Especially if they are used in concert, bluegill pectoral fins and median fins can produce a sufficient stabilizing torque.

Bluegill and other fish may also be able to minimize that cost by pitching. If the COM and COB are displaced along the long axis of the fish, pitching head up or head down would vertically align the COM and COB, which would minimize the destabilizing torques arising from the interaction of the gravitational and buoyant forces. Fishes (He and Wardle 1986; Webb 1993; Wilga and Lauder 1999, 2001), including bluegill (Eidietis et al. 2002) have indeed been observed to change pitch in response to destabilizing forces or to maneuver. Bluegill also swim at a variety of pitching angles at low swimming speeds, which is thought to increase control for stability (Webb 1993). Additionally, larval zebrafish (Zhdanova et al. 2001) and rock bass (Ciancio et al. 2016) have been observed to adopt increased pitch and roll angles respectively while resting, possibly to reduce metabolic costs associated with maintaining an unstable horizontal posture. The ability to increase stability by pitching may also be beneficial to bluegill while feeding. Bluegill are generalist predators (Werner et al. 1978; Ehlinger 1989). They do not swim constantly while feeding but instead, they hover in the water column while searching for prey (Janssen 1982). Hovering at angles pitched from horizontal would increase stability while searching and could reduce the cost of stabilizing an unstable orientation.

## Conclusion and future directions

We found that differences in the volume of the swim bladder do not change the static stability of a bluegill, but the location of the swim bladder can change within a fish depending on body orientation, thus changing the equilibrium orientation of the fish. However, our experiments were performed on dead fish at two opposing pitch extremes. It is still unknown how much changes in swim bladder size or location affects COM/COB dynamics in live bluegill or other fish species during normal behavior.

Understanding the fundamental interaction between ubiquitous environmental forces and the body's inherent material properties is critical to understanding the role that static stability has played in the evolutionary history of fishes. A broad and directed survey of COM and COB locations in fish species with varied body morphologies, swim bladder types, feeding habits, and ecologies is needed to understand why fish bodies tend to be unstable as well as clarify the potentially dynamic nature between the COM and COB in living fish.

## Data Availability

Data for this project is available at: https://dx.doi.org/10.25833/rt3z-7353
